# *Bartonella* spp. in Rats and Zoonoses, Los Angeles, California, USA

**DOI:** 10.3201/eid1804.110816

**Published:** 2012-04

**Authors:** Vijay A.K.B. Gundi, Sarah A. Billeter, Michael P. Rood, Michael Y. Kosoy

**Affiliations:** Centers for Disease Control and Prevention, Fort Collins, Colorado, USA (V.A.K.B. Gundi, S.A. Billeter, M.Y. Kosoy);; Los Angeles County Department of Public Health, Baldwin Park, California, USA (M.P. Rood)

**Keywords:** Bartonella, bacteria, genetic diversity, gltA, rats, Rattus norvegicus, zoonoses, Los Angeles

## Abstract

*Bartonella* spp. were detected in rats (*Rattus norvegicus*) trapped in downtown Los Angeles, California, USA. Of 200 rats tested, putative human pathogens, *B. rochalimae* and *B. tribocorum* were found in 37 (18.5%) and 115 (57.5%) rats, respectively. These bacteria among rodents in a densely populated urban area are a public health concern.

*Bartonella* spp. are vector-borne bacteria associated with an increasing array of emerging zoonotic infections in humans and animals (*1*). Some *Bartonella* spp. are widely distributed among small mammals in the United States and potentially cause human health concerns because these bacteria may be associated with human diseases (*2*). However, limited surveys have been conducted to identify infectious agents involved in zoonotic infections in rodents within urban areas of the United States (*3*). Norway rats (*Rattus norvegicus*) have been shown to harbor several *Bartonella* species, including *B. tribocorum*, *B. elizabethae*, *B. rattimassiliensis*, *B. phoceensis*, *B. queenslandensis*, and a strain closely related to *B. rochalimae* (*3**–**6*), of which *B. elizabethae*, *B. rochalimae*, and *B. tribocorum* were implicated in human diseases (*7**–**11*).

Our study had 3 purposes. The first purpose was to investigate prevalence of *Bartonella* spp. infections in rodent populations in downtown Los Angeles, California, USA. The second purpose was to evaluate genetic diversity of *Bartonella* spp. in blood of urban rats by analyzing variations of the citrate synthase (*gltA*) gene. The third purpose was to compare rates of detection of *Bartonella* spp. infections in rats between culture and molecular assays.

## The Study

Rats were sampled during 2003–2007 at 16 sites in downtown Los Angeles in a rodent management/disease surveillance program. The rats were captured by using Tomahawk live traps (Tomahawk Live Trap Co., Hazelhurst, WI, USA) and anesthetized with CO_2_. Blood samples were obtained by cardiac puncture, transferred to sterile cryovials, placed on dry ice, and preserved at −70°C until testing at the *Bartonella* Laboratory of the Centers for Disease Control and Prevention (Fort Collins, CO, USA).

Genomic DNA was extracted from animal blood according to the blood protocol of the QIAamp DNA Mini Kit (QIAGEN, Valencia, CA, USA). Primers CS140f and CS443f (*12*) and CS781f and CS1137n (*13*), including newly designed primers CS120f-5′-TTTCACTTATGATCCTGGCTT-3′ and CS1210r-5′-GATCYTCAATCATTTCTTTCCA-3′, which amplify DNA fragments ranging from 380 to 1,091 bp encompassing a 327-bp–specific zone (between bp positions 801 and 1127) of the *gltA* gene (*14*), were used in different combinations.

Eight PCRs were performed for each sample. PCR products were purified by using the QIAquick PCR Purification Kit (QIAGEN, Germantown, MD, USA). The same primers were used for DNA sequencing. The obtained DNA sequences were trimmed to 327 bp between positions 801 and 1127 because most *Bartonella* spp. *gltA* sequences available in GenBank are limited to this size.

Details of procedures used for isolation of *Bartonella* spp. from mammalian blood have been described (*2*). Rat blood was diluted 1:4 in brain–heart infusion medium containing 5% fungizone and pipetted onto heart infusion agar plates containing 10% rabbit blood. Plates were incubated aerobically at 35°C in an atmosphere of 5% CO_2_ for <4 weeks and monitored for bacterial growth at least 1× per week after initial plating. Colonies were subpassaged onto fresh agar plates until a pure culture, free from contamination, was obtained.

Analysis of DNA sequences and phylogenetic relationships was performed by using MEGA4 (www.megasoftware.net/) and the neighbor-joining method with the Kimura 2-parameter distance model. Stability of inferred phylogeny was assessed by using bootstrap analysis of 1,000 randomly generated trees. DNA sequences obtained in this study were deposited in GenBank under accession nos. JF429450–JF429625.

A total of 200 *R. norvegicus* rats were trapped in 16 sites in downtown Los Angeles. *Bartonella* DNA was detected in 135 (67.5%) of 200 rat blood samples. PCR-positive blood samples were subsequently cultured for viable *Bartonella* organisms. Fifty-nine (43.7%) of 135 blood samples were confirmed as bacteremic for bartonellae by culturing.

**Figure A1:**
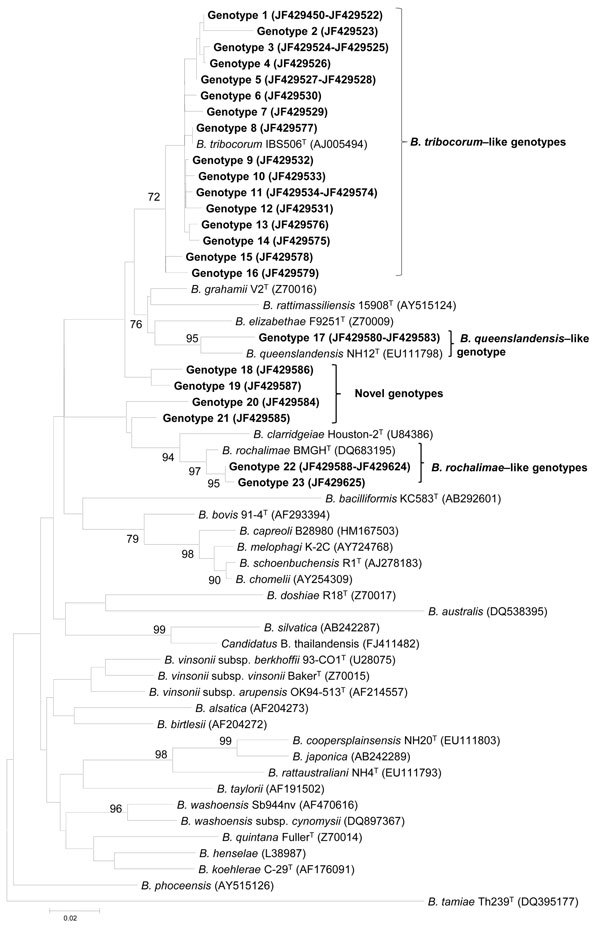
Phylogenetic classification of *Bartonella* spp. genotypes detected in *Rattus norvegicus* rats in downtown Los Angeles, California, USA, on the basis of citrate synthase (*gltA*) gene sequences. The phylogram was constructed by using the neighbor-joining method with the Kimura 2-parameter distance model. Only bootstrap values >70% obtained from 1,000 replicates are given. GenBank accession numbers for reference sequences are indicated in parentheses. Scale bar indicates nucleotide substitutions per site. **Boldface** indicates genotypes identified in rats.

A total of 176 sequences were obtained either directly from blood samples (117 sequences) or from pure cultures (59 sequences). Among 176 *gltA* sequences, 23 genotypes with >1 nt difference were found, and sequence similarity between genotypes ranged from 85.3% to 99.7%. These 23 genotypes were closely related to *B. tribocorum* (n = 16), *B. rochalimae* (n = 2), *B. queenslandensis* (n = 1), or 4 potentially novel genotypes. A total of 130 DNA sequences (JF429450–JF429579) obtained from 115 rats were grouped into 16 genotypes (1–16). These genotypes showed 98.2%–99.7% sequence similarity with each other and were genetically related to *B. tribocorum* IBS506^T^ (AJ005494) (97.9%–100% identity). All 16 genotypes clustered with *B. tribocorum* with a high bootstrap value, as shown in the phylogenetic tree (Figure A1).

Two genotypes (22 and 23) from 38 sequences (JF429588–JF429625) obtained from 37 rats had a similarity between 98.5% and 98.8% to *B. rochalimae* BMGH (DQ683195). These 2 genotypes joined with *B. rochalimae* with a high bootstrap branching pattern in phylogenetic analysis (Figure A1). The *B. queenslandensis* group consisted of a single genotype (genotype 17) from 4 sequences (JF429580–JF429583) from 4 rats. This genotype demonstrated a sequence similarity to *B. queenslandensis* AUST/NH12^T^ (EU111800) of 99.0% for the *gltA* gene (Figure A1).

## Conclusions

In this study, 3 novel *Bartonella* genogroups identified in 4 *R. norvegicus* rats were not genetically related to any known *Bartonella* spp. and might represent novel *Bartonella* spp. Genotypes 18 (JF429586) and 19 (JF429587) formed a *Bartonella* genogroup with 95% similarity to *B. tribocorum* as the closest species. Likewise, genotypes 20 (JF429584) and 21 (JF429585) have 91.4% and 92.6% sequence identities, respectively, with *B. clarridgeiae,* which is their closest related species (Figure A1).

Of 135 PCR-positive blood samples, 59 yielded cultures of 3 *Bartonella* spp.: *B. tribocorum* (n = 54), *B. rochalimae* (n = 4), and *B. queenslandensis* (n = 1). The number of CFU per 100 µL of blood varied: 800–1,400 CFU for *B. tribocorum*, 160–240 CFU for *B. rochalimae*, and 100 CFU for *B. queenslandensis*. Although 4 rats were positive by PCR for *B. queenslandensis*, this organism was cultured from only 1 of these animals.

Our study reports detection and identification of *Bartonella* spp. in urban rats in downtown Los Angeles, California. We demonstrated that *R. norvegicus* in downtown Los Angeles can serve as reservoirs of several *Bartonella* spp., such as *B. rochalimae*, *B. tribocorum*, *B. queenslandensis*, and possibly 3 additional novel species. Some genotypes identified in rats showed a high level of similarity (<98.8%) with a *B. tribocorum* isolate obtained from a febrile patient in Thailand (GenBank accession no. GQ225706) (*11*). Another species (*B. rochalimae*) was isolated from a patient with splenomegaly who had traveled to South America (*14*). This bacterium has also been isolated from dogs, foxes, rats, shrews, gerbils, and raccoons, suggesting that multiple reservoirs may be involved in maintenance of this species. One *Bartonella* genotype found in 4 rats in this study was 99.0% similar to *B. queenslandensis* (GenBank accession no. EU111800), which was originally isolated from *R. fuscipes* rats in Australia (*15*).

Because most identified *Bartonella* species have been reported as human infectious agents elsewhere, the finding that they were circulating among rodents in a densely populated urban area is of serious public health concern. In this context, further studies should be conducted on a larger collection of rodents and clinical human samples to determine evolutionary, genetic, and pathogenic relationships.
